# Carcinoma of the breast during pregnancy and lactation.

**DOI:** 10.1038/bjc.1968.78

**Published:** 1968-12

**Authors:** P. M. Rissanen


### CARCINOMA OF THE BREAST DURING PREGNANCY AND LACTATION

PENTTI M. RISSANEN

*From the Radiotherapy Clinic, University Central Hospital, Helsinki, Finland*

Received for publication June 21, 1968

COINCIDENT pregnancy and carcinoma of the breast is relatively rare. The incidence reported in the literature ranges from 0·43 per cent (Devitt *et al.*, 1964) to 4 per cent (Helman and Bennett, 1963). Most of the materials presented in the literature comprise a couple of cases (*e.g.* Robinson, 1965; Donegan, 1967). Somewhat larger materials and reviews of the literature have been published, as for example by Westberg (1946), Adair (1953), White (1954, 1955), Holleb and Farrow (1962), Miller (1962), Rosemond (1964) and Peters and Meakin (1965). It was generally held earlier that the cases in which carcinoma of the breast is complicated by pregnancy are rather hopeless (*e.g.* Bromeis, 1939; Kettunen, 1946). More recently however, a more optimistic view has been expressed in the literature (*e.g.* Westberg, 1946; Brooks and Proffitt, 1949; Devitt *et al.*, 1964; Schumacher, 1964; Peters and Meakin, 1965). The combination of mammary carcinoma and pregnancy is nevertheless unusual enough to warrant the publication of further experience.

### MATERIAL AND METHODS

The material consisted of 33 patients in whom histologically verified carcinoma of the breast was diagnosed during pregnancy or lactation and who were treated in 1940–61 at the Radiotherapy Clinic, University Central Hospital, Helsinki. This total was about 0·9 per cent of all mammary carcinomas treated at the clinic during the same period. Thirty-one of the patients in the series developed breast cancer during pregnancy and 2 during lactation, both the latter within 3 months of parturition. Three of the patients had another child and one had 2 deliveries later, 1–10 years after treatment for carcinoma of the breast.

The youngest patient was 25 on admission and the oldest 42; the mean age of the series was 34·6 years. The first symptom was invariably a mass in the mammary gland. The duration of symptoms before admission varied from 2 weeks to 2 years, mean 8 months. Some patients had thus felt a mass in the breast *before* the beginning of pregnancy without reacting to it in that phase.

Nineteen of the tumours were in the right and 13 in the left breast. The condition was bilateral in 1 case. The tumour was localised in the lateral half of the breast in 20 cases, in the medial part in 10 cases and centrally in 3. The tumour size varied from 3 cm. to a massive tumour about 15 cm. in diameter. Metastatic lymph nodes in the axilla were established on admission in 25 cases, *i.e.* 75 per cent.

The tumours were distributed into different groups according to the TNM system (U.I.C.C. Publication, 1960) which is in general use today for classification of cancer of the female breast (T = tumour, N = regional lymph nodes, M =

distant metastases). There were no cases of $T_1$, 17 of $T_2$, 15 of $T_3$ and 1 case of $T_4$. The material was distributed as follows on the basis of clinical classification:

| | | |
|---|---|---|
| Stage I | . | 5 cases |
| Stage II | . | 10 ,, |
| Stage III | . | 13 ,, |
| Stage IV | . | 5 ,, |
| Total | . | 33 cases |

Sixteen of the patients in whom mammary carcinoma was established during pregnancy were in the first trimester, 3 in the second and 12 in the third trimester. The cases of different stages were accordingly distributed as shown in Table I.

TABLE I.—*Mammary Carcinomas Established in Different Stages of Pregnancy*

| Stage | I | II | III | IV | Total |
|---|---|---|---|---|---|
| First trimester | 3 | 7 | 5 | 1 | 16 |
| Second trimester | — | 1 | 2 | — | 3 |
| Third trimester | 2 | 1 | 6 | 3 | 12 |
| Total | 5 | 9 | 13 | 4 | 31 |

The distribution according to the histological picture was:

| | |
|---|---|
| Adenomatous carcinoma | 11 |
| " Solid " carcinoma | 9 |
| Scirrhous carcinoma | 3 |
| Colloid carcinoma | 1 |
| Anaplastic carcinoma | 2 |
| Carcinoma (not otherwise specified) | 7 |
| Total | 33 |

No classification into different degrees of malignancy on the basis of the histological picture was performed.

The pregnancy was interrupted in 8 cases; 1 of these was in the second trimester, all the other patients were in the first trimester. Pregnancy was interrupted before the institution of therapy or in course of it in 2 cases and after therapy in 6 cases. Distribution of the abortions by the clinical stage of the tumour was as follows: Stage I: 1 case, Stage II: 3 cases, Stage III: 3 cases and Stage IV: 1 case. The various therapeutic methods employed are shown in Table II. Six patients underwent in addition either surgical or roentgen castration primarily; 2 of them were of Stage I, 1 of Stage II, 2 of Stage III and 1 of Stage IV.

TABLE II.—*The Therapeutic Measures Employed*

| | Stage I | Stage II | Stage III | Stage IV |
|---|---|---|---|---|
| Simple mastectomy + radiotherapy postop. | 2 | — | — | — |
| Radical mastectomy + radiotherapy postop. | 2 | 7 | 7 | 2 |
| Radical mastectomy + radiotherapy preop. and postop. | 1 | 3 | 5 | — |
| Radical mastectomy + radiotherapy preop. | — | — | 1 | — |
| Radiotherapy alone | — | — | — | 2 |
| No specific treatment | — | — | — | 1 |

Preoperative roentgen therapy consisted of *c.* 1400 R for 3–4 weeks from two fields to the breast and from two fields to the corresponding axilla. The treatment values were generally 180–250 kv, 10–15 mA, 0·5 Cu, FSH 50 cm. The supraclavicular fossa, the corresponding axilla and the operative area of the breast were treated postoperatively for 4–5 weeks with a dosage of 1400–2800 R. The 2 patients who were given radiotherapy alone received only a small palliative dose, whereupon treatment had to be discontinued on account of the patients' poor general condition. In one Stage IV case no specific treatment was possible because of the patient's extremely poor general condition.

### RESULTS

The follow-up period was 6–28 years from the start of therapy. Twenty-one patients died during the follow-up period, all of them of mammary carcinoma or its metastases; 12 patients are still alive. Thirty-three cases were followed up for over 5 years, 28 for over 10 years, 20 for over 15 years and 12 for over 20 years. Table III shows the survival rate for the total material. The survival rate according to the various stages is given in Table IV.

TABLE III.—*Survival Rate of the Total Series*

| 5 years | 10 years | 15 years | 20 years |
|---|---|---|---|
| 14/33 | 13/28 | 10/20 | 5/12 |

TABLE IV.—*Survival Rates for the Different Clinical Stages*

| | 1 year | 3 years | 5 years | 10 years | 20 years |
|---|---|---|---|---|---|
| Stage I | 4/5 | 4/5 | 4/5 | 4/5 | 1/1 |
| Stage II | 10/10 | 8/10 | 8/10 | 7/9 | 3/4 |
| Stage III | 10/13 | 3/13 | 2/13 | 2/9 | 1/3 |
| Stage IV | 1/5 | 0/5 | 0/5 | 0/5 | 0/4 |

The 5-year survival rate for the total series was 43 per cent. But analysed according to the different clinical stages, the 5-year rate was 80 per cent for the Stage I cases, 80 per cent for Stage II, 15 per cent for Stage III and 0 per cent for the patients in Stage IV of the disease. One of the patients in Stage IV lived for 1 year 3 months; the rest died within 3 months of admission.

Abortions were performed on 8 patients. Two of them were alive 10–15 years after carcinoma therapy ; one had carcinoma of Stage I and the other of Stage II. The pregnancy was interrupted in the first trimester in both these cases. All the other patients died 3 months–6 years after treatment. One of them was of Stage II, 4 of Stage III and one of Stage IV.

Four of the patients had further pregnancies 1–10 years after treatment for carcinoma of the breast. One of these cases involved a primary tumour of Stage I, 1 of Stage II and 2 of Stage III. One patient in Stage III of the disease who was delivered a year after primary therapy died from metastases $3\frac{1}{2}$ years after primary therapy for carcinoma; all the other 3 patients are alive and free of symptoms 20–24 years after primary therapy.

Of the patients who underwent either surgical or roentgen castration primarily, those of Stage I (2) are alive, while the patients with Stage II–IV of the disease all died 3 months–1 year 3 months after therapy.

### DISCUSSION

Pregnancy as a complication of carcinoma of the breast is fairly rare. The present series represented 0·9 per cent of all mammary carcinomas diagnosed during the same period, an incidence which corresponds with most recent reports. In contrast to the great pessimism voiced in earlier studies (e.g. Bromeis, 1939; Kettunen, 1946) more recent reports suggest that the prognosis of pregnant women with carcinoma of the breast is not essentially different from the general prognosis for young women with mammary carcinoma (e.g. Westberg, 1946; Devitt *et al.*, 1964; Rosemond, 1964; Schumacher, 1964; Peters and Meakin, 1965; Robinson, 1965). On the other hand, mammary carcinoma may grow unusually rapidly during pregnancy, as has been suggested in some experimental (Bromeis, 1939; McCormick and Moon, 1965) and clinical observations. Metastasis to the regional lymph nodes is probably also abnormally rapid in this phase. For instance, Harrington (1937) established metastases in the axillary lymph nodes in 84·7 per cent of his cases, Montgomery (1961) in 74 per cent, and Holleb and Farrow (1962) in 72 per cent. Metastases in the corresponding axilla were established in 75 per cent of the present cases. Donegan (1967), among other authors, encountered axillary metastases in 56·7 per cent of a mammary carcinoma material of non-pregnant women.

Considerable changes occur in the mammary gland even normally in the course of pregnancy. This is probably why patients with breast carcinoma during pregnancy often seek medical advice later than in normal circumstances. Several authors, *e.g.* Westberg (1946) and Bunker and Peters (1963), have reported that these patients come for treatment an average of 2 months later than normally. This makes it understandable that the disease progresses readily. And it is the degree of spread of the disease that is decisive for the prognosis. In cases in which there were no metastases, Harrington (1937) obtained a 5-year survival rate of 61·5 per cent and White (1955) 72·8 per cent. The corresponding survival figures for the metastasising cases were only 5·7 and 6·3 per cent, respectively.

The 5-year survival rate for the total series in the present work was 43 per cent, but 4 patients out of 5 (80 per cent) with carcinoma of Stage I and 8 out of 10 (80 per cent) in Stage II were alive after 5 years. Only 15 per cent of the patients in Stage III and none of those in Stage IV survived. In considering the 5-year survival rate it must be remembered that the majority of the cases (18/33) were patients with far-advanced carcinoma of Stages III–IV. The material is relatively small, of course, which precludes far-reaching conclusions, but everything suggests that if patients with carcinoma of the breast that has begun during pregnancy are treated while the disease is in the initial phases (Stages I–II) the prognosis is good on the whole and hardly worse than the prognosis for mammary carcinoma in young females. Brooks and Proffitt (1949) went as far as to suggest the possibility of a better prognosis. On the other hand, the prognosis is poor in the more advanced cases, Stages III–IV. Thus, it seems obvious that if mammary carcinoma develops during pregnancy the earliest possible diagnosis is the crux of the problem when seeking to improve the therapeutic results.

There is no point with such a small series in comparing the different therapeutic methods. However, the same guide lines as are applicable in the treatment of ordinary mammary carcinoma are valid here.

Interruption of pregnancy as a therapeutic measure for carcinoma of the breast has been discussed widely. In a small mixed group Adair (1953) obtained a higher survival rate when termination of pregnancy was included as part of the therapy. A similar indication was reported by Holleb and Farrow (1962). Montgomery (1962) recommended abortion in the early phase of pregnancy. But many other authors, especially in recent times, have stated that there is not sufficient statistical evidence of the clinical significance of termination of pregnancy (*e.g.* White, 1954; Miller, 1962; Schumacher, 1964) for the course of the disease, and that for this reason abortion cannot be regarded as being of indisputable significance in the treatment of the disease. Peters and Meakin (1965) stated that there is to date no medical indication for interrupting a pregnancy during any phase. Eight of the present cases underwent interruption of pregnancy. Of these patients, one with Stage I and 2 with Stage II of the disease were alive after 5 years; one patient with Stage II, 3 with Stage III and one with Stage IV died during this time. The material was too small to evaluate the significance of abortion (Table V), but it seems that it has not been possible to prove that abortion

TABLE V.—*Correlation of Mammary Carcinomas Diagnosed During Pregnancy with Pregnancy and 5-Year Survival*

| | 5-year survival | |
|---|---|---|
| Clinical stage | Pregnancy interrupted | Pregnancy not interrupted |
| St I . . . | 1/1 | 3/4 |
| St II . . . | 2/3 | 5/6 |
| St III . . . | 0/3 | 2/10 |
| St IV . . . | 0/1 | 0/3 |
| Total . | 3/8 | 10/23 |

has a beneficial effect on the end result. Thus, the decision to intervene must be weighed in each individual case with reference to many other standpoints. However, the most important factor is early diagnosis and the most urgent surgical measure possible for the removal of the tumour primarily, always regardless of the stage of the pregnancy, and possibly combined with radiotherapy.

Surgical or roentgenologic castration was performed as a primary measure on 6 patients; 2 patients with Stage I of the disease were alive after 5 years, the others died during this period. Because of the small number of cases, no conclusions can be drawn regarding the therapeutic value of this intervention in the more advanced cases. Many authors (*e.g.* Rosemond 1954) oppose prophylactic castration because its benefit has not yet been proved in larger series.

### SUMMARY

The series comprised 33 patients with histologically verified mammary carcinoma during pregnancy or lactation who were treated in 1940–61. This total was 0·9 per cent of all carcinomas of the breast treated at the same clinic during the period. The mean age of the patients was 34·6 years. The clinical classification by stage (TNM system) was: Stage I—5 cases, Stage II—10 cases, Stage III—13 cases, Stage IV—5 cases. Seventy-five per cent of the patients had metastases in the homolateral axillary glands. The commonest therapeutic measure was radical operation combined with postoperative radiotherapy. In addition, termination of pregnancy was performed in 8 cases and primary castration in 6.

The 5-year survival rate for the total series was 43 per cent, but it was 80 per cent for patients in Stages I and II of the disease.

This investigation was supported by a grant from the Sigrid Jusélius Foundation and the President J. K. Paasikivi's Foundation for Cancer Research, Helsinki, Finland.

### REFERENCES

ADAIR, F.—(1953) *Surg. Clins N. Am.*, **33**, 313.
BROMEIS, H.—(1939) *Dt. Z. Chir.*, **252**, 294.
BROOKS, B. AND PROFFITT, J. W.—(1949) *Surgery, St Louis*, **25**, 1.
BUNKER, M. L. AND PETERS, M. V.—(1963) *Am. J. Obstet. Gynec.*, **85**, 312.
DEVITT, J. E., BEATTIE, W. G. AND STODDART, T. G.—(1964) *Can. J. Surg.*, **7**, 124.
DONEGAN, W. H.—(1967) In 'Cancer of the Breast. Major Problems in Clinical Surgery'. Edited by Spratt and Donegan. Philadelphia (Saunders Co.).
HARRINGTON, S. W.—(1937) *Ann. Surg.*, **106**, 690.
HELMAN, P. AND BENNETT, M. B.—(1963) *S. Afr. med. J.*, **37**, 1236.
HOLLEB, A. I. AND FARROW, J. H.—(1962) *Surgery Gynec. Obstet.*, **65**, 115.
International Union Against Cancer (1960). Committee on Clinical Stage Classification and Applied Statistics. 'Malignant Tumours of the Breast.'
KETTUNEN, K.—(1946) *Nord. méd.*, **31**, 1747.
MCCORMICK, G. M. AND MOON, R. C.—(1965) *Br. J. Cancer.*, **19**, 160.
MILLER, H. K.—(1962) *Am. J. Obstet. Gynec.*, **83**, 607.
MONTGOMERY, T. L.—(1961) *Am. J. Obstet. Gynec.*, **81**, 926.
PETERS, V. M. AND MEAKIN, J. W.—(1965) In 'Progress in Clinical Cancer', Vol. I. Edited by Irving M. Arrel. New York (Grune & Stratton Inc.).
ROBINSON, D. W.—(1965) *Am. J. Obstet. Gynec.*, **92**, 658.
ROSEMOND, G. P.—(1964) *N.Y. Acad. Sci.*, **114**, 851.
SCHUMACHER, J.—(1964) *Grace Hosp. Bull.*, **42**, 36.
WESTBERG, S. V.—(1946) *Acta Obstet. Gynec. scand.*, **4**, 1.
WHITE, T. T.—(1954) *Ann. Surg.*, **139**, 9.—(1955) *Surgery Gynec. Obstet.*, **100**, 661.


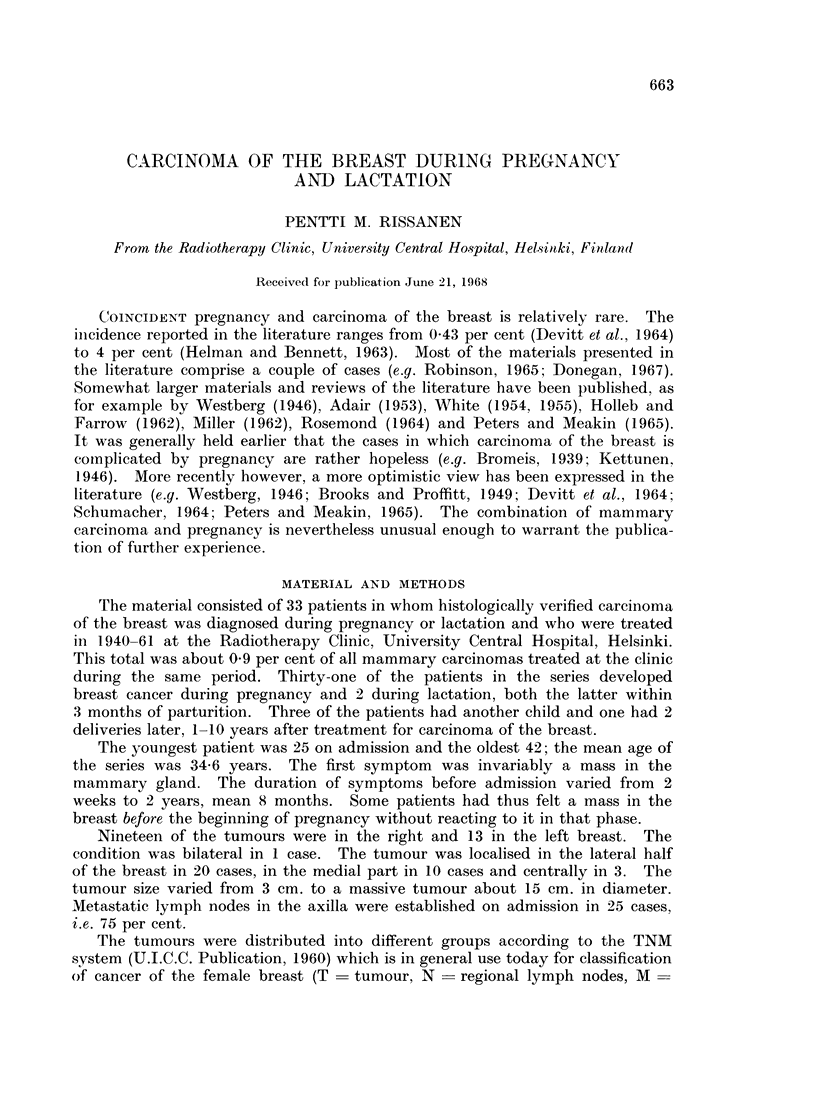

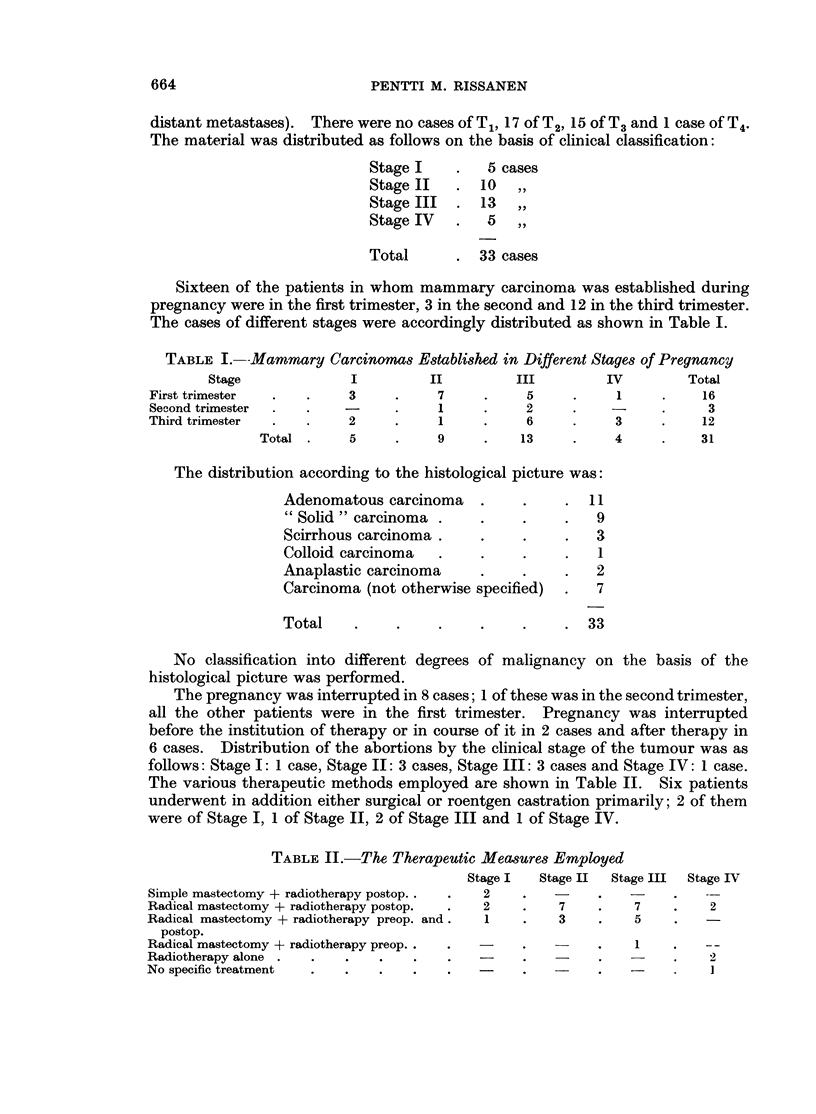

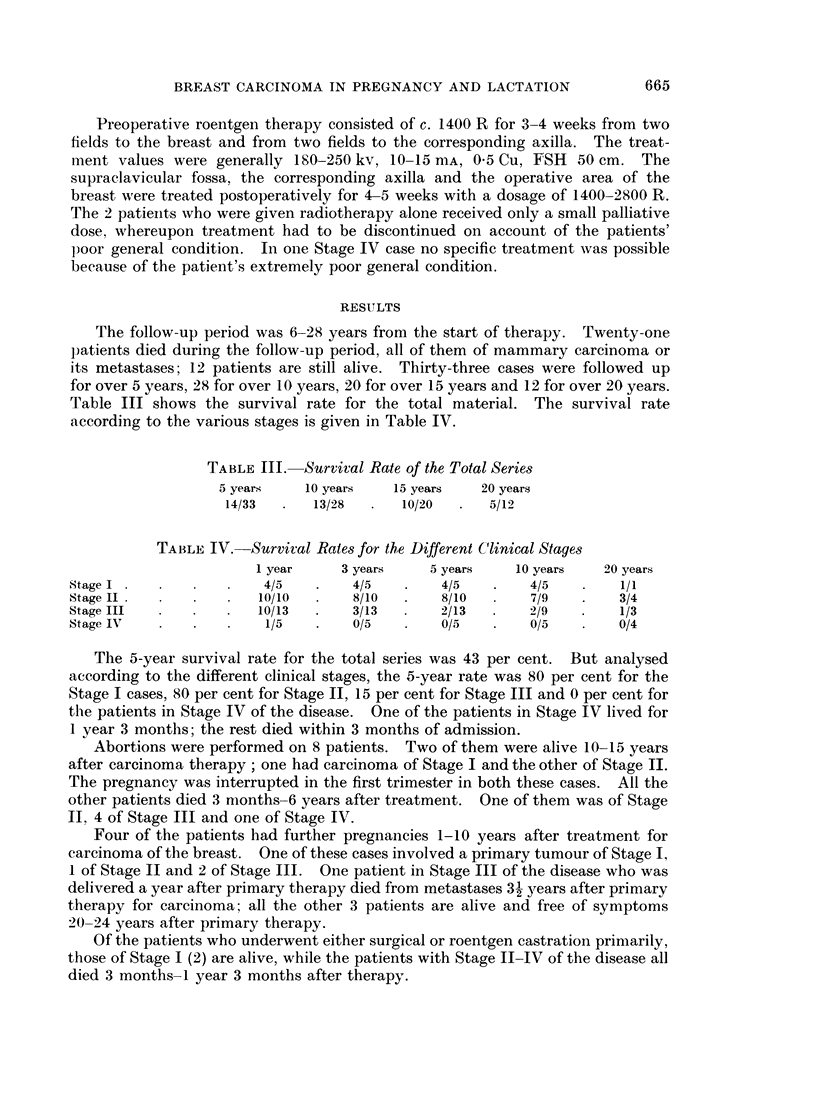

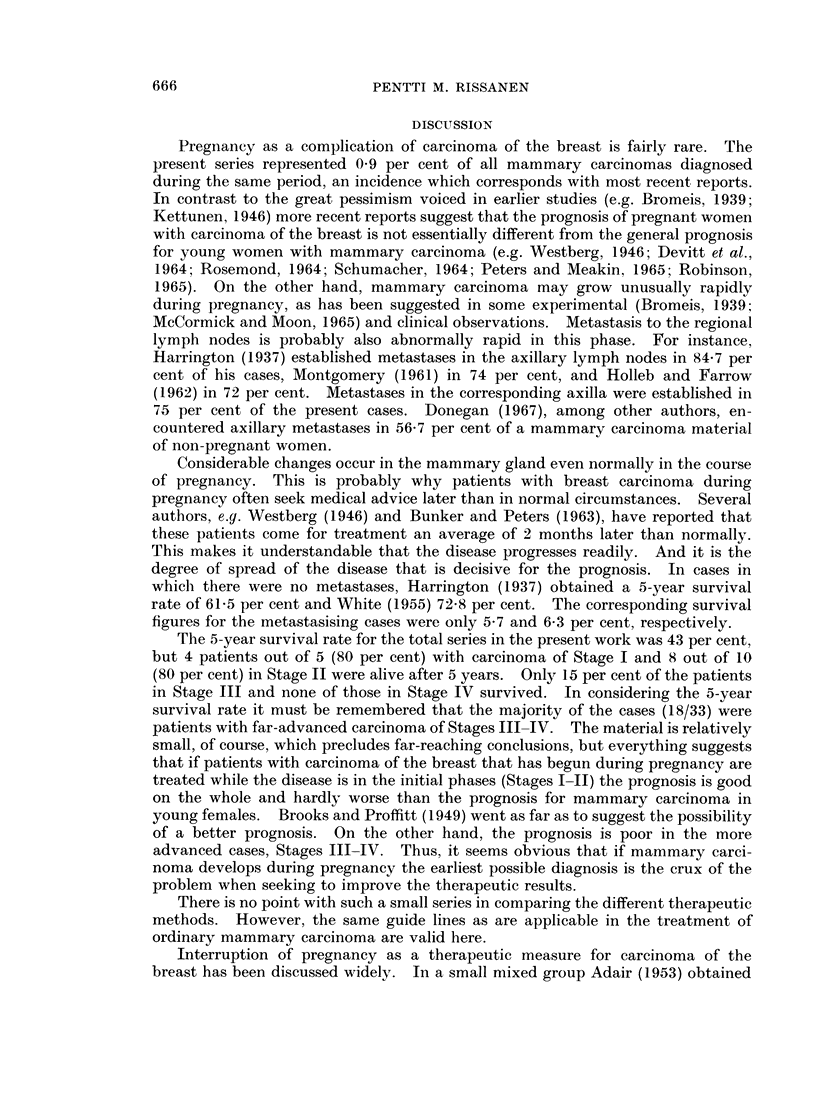

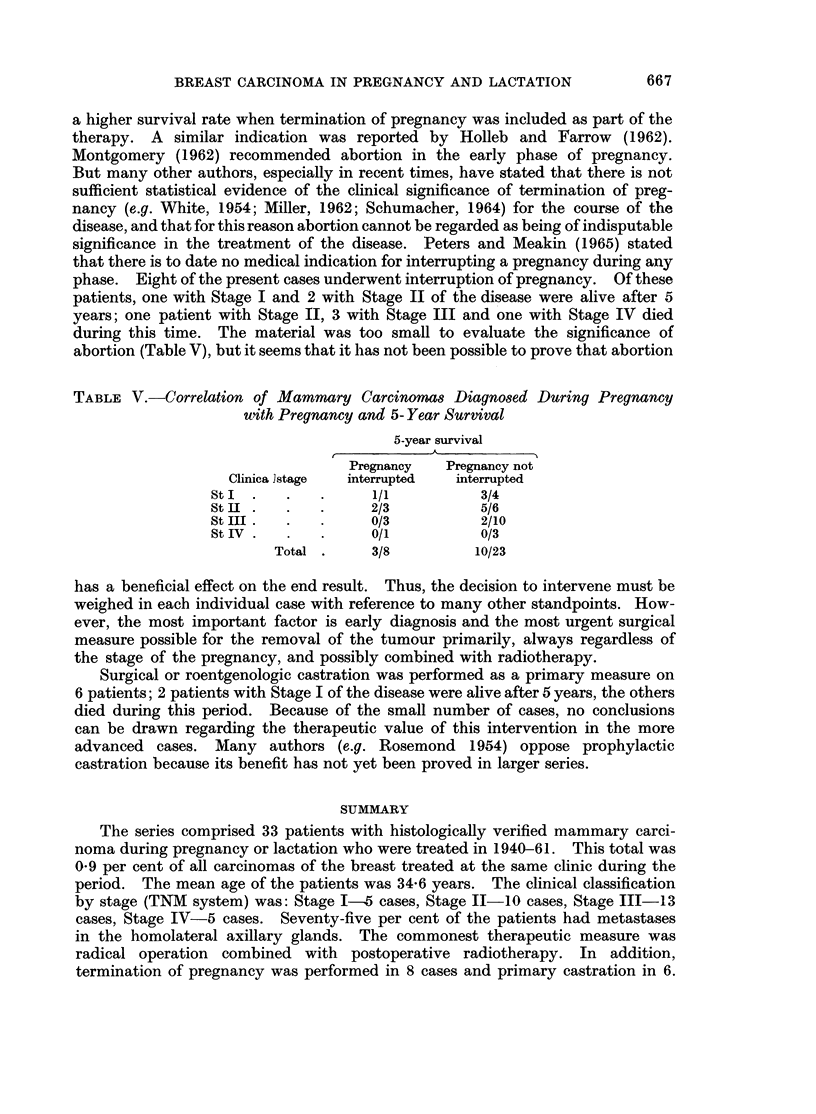

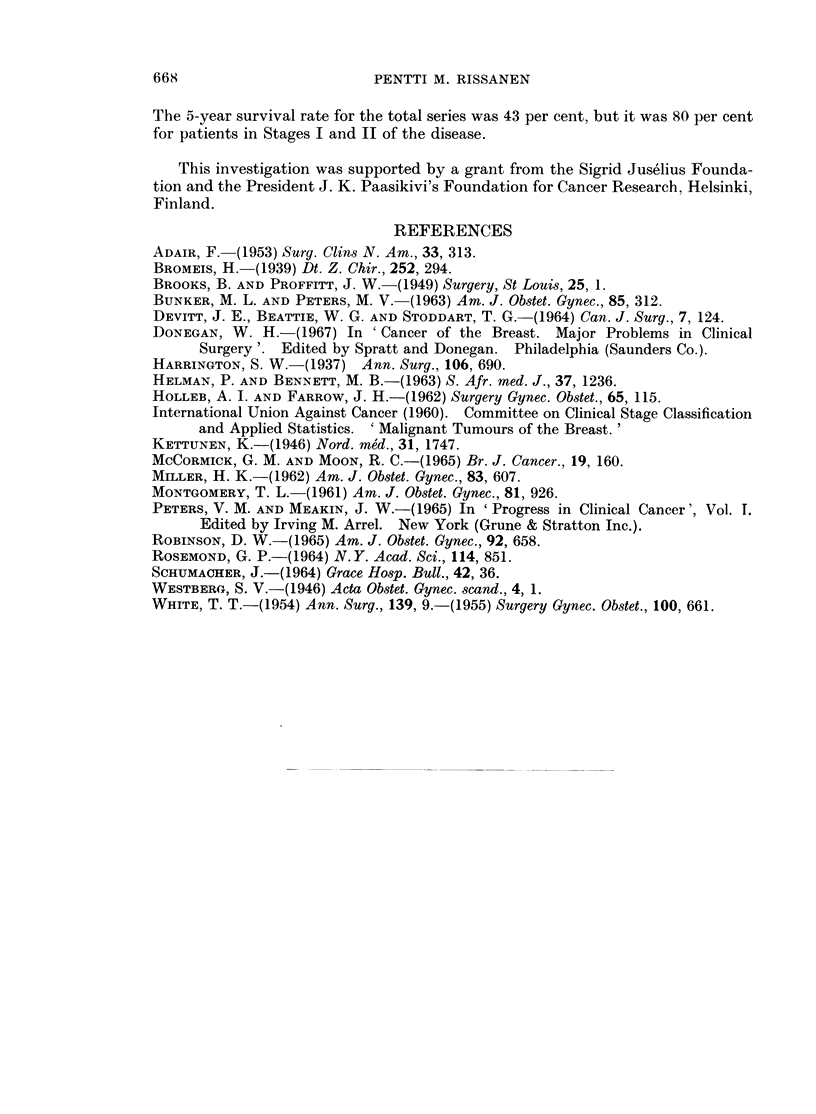


## References

[OCR_00317] DEVITT J. E., BEATTIE W. G., STODDART T. G. (1964). CARCINOMA OF THE BREAST AND PREGNANCY.. Can J Surg.

[OCR_00322] Harrington S. W. (1937). CARCINOMA OF THE BREAST RESULTS OF SURGICAL TREATMENT WHEN THE CARCINOMA OCCURRED IN THE COURSE OF PREGNANCY OR LACTATION AND WHEN PREGNANCY OCCURRED SUBSEQUENT TO OPERATION (1910-1933).. Ann Surg.

[OCR_00333] MCCORMICK G. M., MOON R. C. (1965). EFFECT OF PREGNANCY AND LACTATION ON GROWTH OF MAMMARY TUMOURS INDUCED BY 7,12-DIMETHLBENZ(A) ANTHRACENE (DMBA).. Br J Cancer.

[OCR_00334] MILLER H. K. (1962). Cancer of the breast during pregnancy and lactation.. Am J Obstet Gynecol.

[OCR_00336] MONTGOMERY T. L. (1961). Detection and disposal of breast cancer in pregnancy.. Am J Obstet Gynecol.

[OCR_00341] ROBINSON D. W. (1965). BREAST CARCINOMA ASSOCIATED WITH PREGNANCY; OBSERVATIONS ON 1,128 CASES OF BREAST CARCINOMA.. Am J Obstet Gynecol.

[OCR_00342] ROSEMOND G. P. (1964). MANAGEMENT OF PATIENTS WITH CARCINOMA OF THE BREAST IN PREGNANCY.. Ann N Y Acad Sci.

